# Genome Sequence of the Lactic Acid Bacterium *Lactococcus lactis* subsp. *lactis* TOMSC161, Isolated from a Nonscalded Curd Pressed Cheese

**DOI:** 10.1128/genomeA.01121-14

**Published:** 2014-11-06

**Authors:** H. Velly, P. Renault, A.-L. Abraham, V. Loux, A. Delacroix-Buchet, F. Fonseca, M. Bouix

**Affiliations:** a Centre National Interprofessionnel de l’Économie Laitière, Paris, France; b INRA, UMR 782 Génie et Microbiologie des Procédés Alimentaires (GMPA), Thiverval-Grignon, France; c INRA, UMR 1319 Micalis, Domaine de Vilvert, Jouy-en-Josas, France; d AgroParisTech, UMR 1319 Micalis, Jouy-en-Josas, France; e INRA, UR 1077 Mathématique, Informatique et Génome (MIG), Domaine de Vilvert, Jouy-en-Josas, France

## Abstract

*Lactococcus lactis* is a lactic acid bacterium used in the production of many fermented foods, such as dairy products. Here, we report the genome sequence of *L. lactis* subsp. *lactis* TOMSC161, isolated from nonscalded curd pressed cheese. This genome sequence provides information in relation to dairy environment adaptation.

## GENOME ANNOUNCEMENT

*Lactococcus lactis* belongs to the group of lactic acid bacteria and is encountered in a wide range of environments, such as animals and plant materials, and even in a great variety of traditional food products (as spontaneous growth) (1, 2). Industrially, this mesophilic and homofermentative bacterium is extensively used as a starter in the production of many fermented foods, and it is particularly used in the dairy industry for cheesemaking. *L. lactis*, which has generally regarded as safe (GRAS) status, is involved in milk acidification by the formation of lactate, proteolysis, and in the synthesis of aromatic compounds, polysaccharides, and bacteriocins, contributing therefore to the taste, texture, and safety of dairy products (3–7).

*L. lactis*, which is by far the best characterized lactic acid bacterium (LAB) with respect to its physiology, metabolic pathways, and regulatory mechanisms, has become the model bacterium for most of the LAB research in biotechnology. The genomes of several *L. lactis* strains have thus been determined, including strains of *L. lactis* subsp. *lactis*, such as IL1403 (8), KF147 (9), and CV56 (10), as well as strains of *L. lactis* subsp. *cremoris*, such as MG1363 (11) and SK11 (12). We report here the genome of *L. lactis* subsp. *lactis* TOMSC161, a strain isolated from the dairy environment and more specifically from a scaled nonscalded curd pressed cheese, and presenting very interesting technological properties (acidifying, proteolytic, and texturing activities).

Sequencing was performed by Imagif (CNRS, Gif-sur-Yvette, France) using HiSeq paired-end sequencing. A total of 20,391,131 reads were used for assembly by using the Velvet and VelvetOptimiser softwares, leading to an average coverage of 730×. A total of 56 large contigs ranging from 1,013 to 431,322 bp in size were generated and annotated using AGMIAL, an integrated bacterial genome annotation platform (13).

The genome of *L. lactis* subsp. *lactis* TOMSC161 consists of 2,603,548 bp, with an average G+C content of 35.09%. It contains 2,686 protein-coding sequences covering 86% of the genome, of which 1,991 were annotated with known biological functions and 695 encode hypothetical proteins. It also harbors 53 tRNA-coding genes and 5 rRNA-coding operons.

Analysis of the genome of *L. lactis* TOMSC161 revealed an adaptation to milk, a nutrient-rich environment. The presence of the complete set of genes involved in lactose and galactose metabolism, as well as the presence of a wide variety of substrate transporters (carbohydrates, peptides, and amino acids) and inversely, the reduction of several amino acid biosynthetic capacities (auxotrophic for glutamate and aspartate) demonstrated this adaptation. However, the genome of *L. lactis* TOMSC161 still contains genes involved in the utilization of certain plant sugars (mannose, mannitol, arabinose, ribose, and xylose) related to the original habitat of *L. lactis*. Furthermore, the analysis showed the presence of genes involved in exopolysaccharide and bacteriocin (e.g., lactococcin) production, which are interesting technological features.

The availability of the genome sequence of *L. lactis* TOMSC161 will allow the powerful application of transcriptomics and proteomics to investigate the metabolic responses and thus the physiological processes involved in the adaptation to different environmental conditions.

### Nucleotide sequence accession numbers.

The genome sequence and annotation of *L. lactis* subsp. *lactis* TOMSC161 have been deposited in the EMBL database under accession numbers CBUJ010000001 to CBUJ010000062.
